# Supplementary material for: Understanding the direct and indirect impacts of disease response phenotypes on chicken coccidiosis epidemiology: A modelling approach
Source: PLoS One. 2026 Mar 5;21(3):e0343712. doi: 10.1371/journal.pone.0343712 (PMC12962546; doi:10.1371/journal.pone.0343712)
Supplement: S1 Table — (DOCX) [file pone.0343712.s001.docx]

**Supplementary Table 1.** States of Each Pair in Experiment I: 5-Oocyst Group (9 pairs)

|  | pair (with birds index) | | | | | | | | | | | | | | | | | |
| --- | --- | --- | --- | --- | --- | --- | --- | --- | --- | --- | --- | --- | --- | --- | --- | --- | --- | --- |
|  | 1 | | 2 | | 3 | | 4 | | 5 | | 6 | | 7 | | 8 | | 9 | |
| Day.start | 5I | 1C | 26I | 19C | I23 | 4C | 6I | 9C | 20I | 16C | I3 | 8C | 2I1 | 30C | 24I | 25C | 27I | 2C |
| 4 | E0 | S0 | E0 | S0 | E0 | S0 | E0 | S0 | E0 | S0 | E0 | S0 | E0 | S0 | E0 | S0 | E0 | S0 |
| 5 | I0 | S0 | I0 | S0 | I0 | S0 | I0 | S0 | I0 | S0 | E0^*^ | S0 | I0 | S0 | I0 | S0 | I0 | S0 |
| 6 | I0 | S0 | I0 | E0 | I0 | S0 | I0 | E0 | I0 | E0 | E0 | S0 | I0 | S0 | I0 | E0 | I0 | S0 |
| 7 | S1 | S0 | I0 | E0 | S1^**^ | S0 | I0 | E0 | I0 | E0 | I0 | S0 | S1^**^ | S0 | I0 | E0 | I0 | E0 |
| 8 | E1^**^ | S0 | I0 | E0 | E1^**^ | E0 | I0 | E0 | I0 | E0 | S1^**^ | E0 | E1^**^ | E0 | I0^*^ | E0 | S1^**^ | E0 |
| 9 | E1^**^ | E0 | I0^*^ | E0 | E1^**^ | E0 | I0^*^ | E0 | I0^*^ | E0 | E1^**^ | E0 | E1^**^ | E0 | I0^*^ | E0 | E1^**^ | E0 |
| 10 | E1^**^ | E0 | I0^*^ | I0 | E1^**^ | E0 | I0^*^ | I0 | I0 | I0 | E1^**^ | E0 | E1^**^ | E0 | I0 | I0 | E1^**^ | E0 |
| 11 | I1 | E0 | I0 | I0^*^ | I1 | E0 | I0 | I0 | I0 | I0 | I1 | E0 | I1 | E0 | I0 | I0 | I1 | I0 |
| 12 | I1 | E0 | I0 | I0 | I1 | I0 | I0 | I0 | I0 | I0 | I1 | I0 | I1 | I0 | I0 | I0 | I1 | I0 |
| 13 | I1 | I0 | I0 | I0 | I1 | S1^**^ | I0 | I0 | I0 | I0 | I1 | I0 | I1 | I0 | I0 | I0 | I1 | I0 |
| 14 | I1 | I0 | I0 | I0 | I1 | E1^**^ | I0 | I0 | I0^*^ | I0 | I1 | I0 | I1 | I0 | I0 | I0 | I1 | I0 |
| 15 | I1 | I0^*^ | I0 | I0 | I1 | E1^**^ | I0 | I0 | I0^*^ | I0 | I1 | I0 | I1 | I0 | I0 | I0 | I1 | I0 |
| 16 | I1 | I0 | I0 | I0 | I1 | I1 | I0 | I0 | I0 | I0 | I1 | I0 | I1 | I0 | I0 | I0 | I1 | I0 |
| 17 | I1 | I0 | S1^**^ | I0 | I1 | I1 | I0 | I0 | I0 | I0 | I1 | I0 | I1 | I0 | I0 | I0 | I1 | I0 |
| 18 | I1 | I0 | E1^**^ | I0 | I1 | I1 | I0 | I0 | I0 | I0 | I1 | I0 | I1^*^ | I0 | I0 | I0 | I1 | I0 |
| 19 | I1 | I0 | E1^**^ | I0 | I1 | I1 | I0 | I0 | I0 | I0 | I1 | I0 | I1 | I0 | I0 | I0 | I1 | I0 |
| 20 | S2 | I0 | E1^**^ | I0 | I1 | I1 | I0 | I0 | I0 | S1 | I1 | I0 | I1 | I0 | I0 | S1 | I1 | I0 |
| 21 | S2 | I0 | E1^**^ | I0 | I1 | I1 | I0^*^ | I0 | I0^*^ | S1 | I1 | I0 | I1 | I0 | I0^*^ | S1 | I1 | S1^**^ |
| 22 | S2^*^ | I0 | I1 | I0 | I1 | I1 | I0 | I0 | I0 | S1^**^ | I1 | I0 | I1 | I0 | I0 | S1^**^ | I1 | E1^**^ |
| 23 | E2^*^ | I0 | I1^*^ | I0 | I1 | I1 | I0 | I0^*^ | I0 | E1^**^ | S2^*^ | S1 | I1 | I0 | I0 | E1^**^ | I1 | E1^**^ |
| 24 | E2 | I0 | I1 | I0 | S2 | I1 | S1^**^ | I0 | I0 | E1^**^ | E2 | S1 | I1 | I0 | I0 | E1^**^ | S2 | E1^**^ |
| 25 | E2 | I0^*^ | I1^*^ | S1^**^ | E2^*^ | I1 | E1^**^ | I0 | I0 | E1^**^ | E2 | S1 | I1 | S1^**^ | S1^**^ | E1^**^ | S2 | E1^**^ |
| 26 | E2 | I0 | I1 | E1^**^ | E2 | I1 | E1^**^ | I0 | I0 | E1^**^ | I2 | S1 | S2 | E1^**^ | E1^**^ | E1^**^ | S2^*^ | E1^**^ |
| 27 | I2 | I0 | I1 | E1^**^ | E2 | I1 | E1^**^ | I0 | I0 | I1 | I2 | S1 | E2 | E1^**^ | E1^**^ | I1 | S2 | E1^**^ |
| 28 | I2 | I0^*^ | I1 | I1 | I2 | I1 | E1^**^ | I0 | I0 | I1 | I2 | S1 | E2 | I1 | I1 | I1 | S2^**^ | E1^**^ |
| 29 | I2 | I0 | I1 | I1 | I2 | I1 | I1 | I0 | I0^*^ | I1 | I2 | S1 | I2 | I1 | I1 | I1 | S2^**^ | E1^**^ |

Susceptible individuals are specified into four classes (S0, S1, S2, S3) represent the first, second, third, and fourth time of being susceptibility, each corresponding to varying levels of immunity and susceptibility. The corresponding exposed/latently infected states are denoted as E0, E1, E2, E3 and the corresponding infectious states are denoted as I0, I1, I2, I3.

^*^ Exception in a continuous period: The state is adjusted to the same state as the adjacent days.

^**^ No data available: The state is inferred from adjacent days.
