# Supplementary material for: Understanding the direct and indirect impacts of disease response phenotypes on chicken coccidiosis epidemiology: A modelling approach
Source: PLoS One. 2026 Mar 5;21(3):e0343712. doi: 10.1371/journal.pone.0343712 (PMC12962546; doi:10.1371/journal.pone.0343712)
Supplement: S2 Table — (DOCX) [file pone.0343712.s002.docx]

**Supplementary Table 2.** States of Each Pair in Experiment II: 50-Oocyst Group (17 pairs)

|  | pair (with birds index) | | | | | | | | | | | | | | | | | |
| --- | --- | --- | --- | --- | --- | --- | --- | --- | --- | --- | --- | --- | --- | --- | --- | --- | --- | --- |
|  | 1 | | 2 | | 3 | | 4 | | 5 | | 6 | | 7 | | 8 | | 9 | |
| Day.start | 518I | 514C | 5I23 | 540C | 5I12 | 559C | 526I | 567C | 547I | 508C | 5I13 | 515C | 507I | 531C | 528I | 601C | 538I | 539C |
| 4 | E0 | S0^**^ | E0 | S0^**^ | E0 | S0^**^ | E0 | S0^**^ | E0 | S0^**^ | E0 | S0^**^ | E0 | S0^**^ | E0 | S0^**^ | E0 | S0^**^ |
| 5 | I0 | S0^**^ | E0^**^ | S0^**^ | E0^**^ | S0^**^ | E0^**^ | S0^**^ | I0 | S0^**^ | I0 | S0^**^ | I0 | S0^**^ | I0 | S0^**^ | I0 | S0^**^ |
| 6 | I0 | S0^**^ | I0 | S0^**^ | I0 | S0^**^ | I0 | S0^**^ | I0 | E0^**^ | S1^**^ | S0^**^ | S1^**^ | S0^**^ | I0 | S0^**^ | I0 | S0^**^ |
| 7 | I0 | E0 | I0 | E0^**^ | I0 | E0^**^ | I0^*^ | E0 | I0 | E0^**^ | E1^**^ | E0^**^ | E1^**^ | E0^**^ | I0 | E0^**^ | I0 | E0^**^ |
| 8 | I0 | E0 | S1^**^ | E0 | I0^*^ | E0 | I0^*^ | E0^*^ | S1^**^ | E0 | E1^**^ | E0 | E1^**^ | E0 | I0 | E0 | I0 | E0 |
| 9 | I0^*^ | E0 | E1^**^ | E0 | I0 | E0 | I0 | E0 | E1^**^ | E0 | E1^**^ | E0 | E1^**^ | E0 | I0^*^ | E0 | I0^*^ | E0 |
| 10 | I0 | E0 | E1^**^ | I0 | I0 | E0 | I0 | I0 | E1^**^ | I0 | E1^**^ | E0 | I1 | E0 | I0^*^ | E0 | I0^*^ | E0 |
| 11 | I0 | I0 | I1 | I0 | I0 | I0 | I0 | I0 | I1 | I0 | I1 | I0 | I1 | I0 | I0 | I0 | I0 | I0 |
| 12 | I0 | I0 | I1 | I0 | I0 | I0 | I0 | I0 | I1 | I0 | I1 | I0 | I1 | I0 | I0 | I0 | I0 | I0^**^ |
| 13 | I0 | I0 | I1 | I0 | I0 | I0 | I0 | I0 | I1 | I0 | I1 | I0 | I1 | S1^**^ | I0 | I0 | I0 | I0 |
| 14 | I0 | I0 | I1 | I0 | I0 | I0 | I0^**^ | I0 | I1^**^ | I0 | I1 | I0 | I1 | E1^**^ | I0 | I0 | I0 | I0 |
| 15 | I0 | I0 | I1 | I0 | I0 | I0 | I0 | I0 | I1^*^ | I0 | I1 | I0 | I1 | E1^**^ | I0 | I0 | I0 | I0 |
| 16 | I0 | I0 | I1 | I0 | I0 | I0 | I0 | I0 | I1 | I0 | I1 | I0 | I1 | I1 | I0^*^ | I0 | I0 | I0 |
| 17 | I0 | I0 | I1 | I0 | I0 | I0 | I0 | I0 | I1 | I0 | I1 | I0 | I1 | I1 | I0 | I0 | I0 | I0 |
| 18 | I0 | I0 | I1 | I0 | I0 | I0 | I0 | I0 | I1 | I0 | I1 | I0 | I1 | I1 | I0 | I0 | I0 | I0^**^ |
| 19 | I0 | I0 | I1 | I0 | I0 | I0 | I0 | I0 | I1 | I0 | I1 | I0 | I1 | I1 | I0 | I0 | I0 | I0 |
| 20 | S1 | I0 | I1 | I0 | S1^**^ | I0 | I0^*^ | I0 | I1 | I0 | I1 | I0 | I1 | I1 | I0 | I0 | I0 | I0 |
| 21 | S1 | I0 | I1 | S1 | E1^**^ | I0 | I0^*^ | I0^*^ | I1 | S1 | I1 | S1 | I1 | I1 | I0 | S1^**^ | I0^*^ | I0 |
| 22 | S1 | I0 | I1 | S1 | E1^**^ | I0 | I0 | I0 | I1 | S1 | I1 | S1 | S2 | I1 | S1 | E1^**^ | I0^*^ | S1^**^ |
| 23 | S1 | S1 | S2 | S1 | E1^**^ | I0 | I0 | S1^**^ | I1 | S1 | I1 | S1 | S2^*^ | I1 | S1 | E1^**^ | I0 | E1^**^ |
| 24 | S1^**^ | S1^**^ | S2^**^ | S1^**^ | E1^**^ | S1^**^ | I0^**^ | E1^**^ | S2^**^ | S1^**^ | I1^**^ | S1^**^ | S2^**^ | S2^**^ | S1^**^ | E1^**^ | S1^**^ | E1^**^ |
| 25 | S1 | S1 | S2 | S1 | I1 | S1 | I0 | E1^**^ | S2 | S1 | I1 | S1 | S2 | E2 | S1 | I1 | S1 | E1^**^ |
| 26 | S1 | S1 | S2 | S1 | S2 | S1 | S1 | I1 | S2 | S1 | I1 | S1 | S2 | E2 | S1 | S2 | S1 | I1 |
| 27 | S1 | S1 | S2 | S1 | S2 | S1 | S1 | I1 | S2 | S1 | I1 | S1 | S2 | I2 | S1 | S2 | S1 | S2 |
| 28 | S1^**^ | S1^**^ | S2^**^ | S1^**^ | S2^**^ | S1^**^ | S1^**^ | S2^**^ | S2^**^ | S1^**^ | I1 | S1^**^ | S2^**^ | S3 | S1^**^ | S2^**^ | S1^**^ | S2 |
| 29 | S1 | S1 | S2 | S1 | S2 | S1 | S1 | S2 | S2 | S1 | S2 | S1 | S2 | S3 | S1 | S2 | S1 | S2 |
| 30 | S1^**^ | S1^**^ | S2^**^ | S1^**^ | S2^**^ | S1^**^ | S1^**^ | S2^**^ | S2^**^ | S1^**^ | S2^**^ | S1^**^ | S2^**^ | S3^**^ | S1^**^ | S2^**^ | S1^**^ | S2^**^ |
|  |  |  |  |  |  |  |  |  |  |  |  |  |  |  |  |  |  |  |
|  | pair (with birds index) | | | | | | | | | | | | | | | |  |  |
|  | 10 | | 11 | | 12 | | 13 | | 14 | | 15 | | 16 | | 17 | |  |  |
| Day.start | 53I3 | 554C | 529I | 542C | 536I | 563C | 557I | 558C | 549I | 506C | 556I | 570C | 564I | 551C | 53I2 | 516C |  |  |
| 4 | I0 | S0^**^ | E0 | S0^**^ | E0 | S0^**^ | E0 | S0^**^ | E0 | S0^**^ | E0 | S0^**^ | E0 | S0^**^ | E0 | S0^**^ |  |  |
| 5 | I0 | S0^**^ | I0 | S0^**^ | I0 | S0^**^ | I0 | S0^**^ | E0 | S0^**^ | I0 | S0^**^ | E0 | S0^**^ | I0 | S0^**^ |  |  |
| 6 | I0 | E0^**^ | I0 | E0^**^ | I0 | S0^**^ | I0 | S0^**^ | I0 | S0^**^ | I0 | E0^**^ | E0 | S0^**^ | I0 | E0^**^ |  |  |
| 7 | I0 | E0^**^ | I0 | E0^**^ | I0 | E0 | S1^**^ | S0 | S1^**^ | S0^**^ | I0 | E0^**^ | E0 | S0^**^ | I0 | E0^**^ |  |  |
| 8 | I0 | E0 | I0 | E0 | I0 | E0 | E1^**^ | E0 | E1^**^ | E0 | I0 | I0 | I0 | S0 | I0^*^ | E0 |  |  |
| 9 | I0 | E0 | I0^*^ | E0 | I0^*^ | E0 | E1^**^ | E0 | E1^**^ | E0 | I0 | I0^*^ | I0^*^ | E0 | I0^*^ | E0 |  |  |
| 10 | I0 | I0 | I0 | I0 | I0 | E0 | I1 | E0 | E1^**^ | E0 | I0 | I0 | I0^*^ | E0 | I0 | I0 |  |  |
| 11 | I0 | I0 | I0 | I0 | I0 | I0 | I1 | E0 | I1 | E0 | I0 | I0 | I0 | I0 | I0 | I0 |  |  |
| 12 | I0 | I0 | I0 | I0 | I0 | I0 | I1 | I0 | I1 | I0 | I0 | I0 | S1^**^ | I0 | I0 | I0 |  |  |
| 13 | I0 | I0 | I0 | I0 | I0 | I0 | I1 | I0 | I1 | I0 | I0 | I0 | E1^**^ | I0 | I0 | I0 |  |  |
| 14 | I0 | I0 | I0 | I0 | I0 | I0 | I1 | I0^**^ | I1 | S1 | I0 | I0 | E1^**^ | I0 | I0 | I0 |  |  |
| 15 | I0 | I0 | I0 | I0 | I0 | I0 | I1 | I0 | I1 | S1 | I0 | I0 | E1^**^ | I0 | I0 | I0 |  |  |
| 16 | I0 | I0 | I0 | I0 | I0 | I0 | I1 | I0 | I1 | E1^**^ | I0 | I0 | I1 | I0 | I0 | I0 |  |  |
| 17 | I0 | I0 | I0 | I0 | I0 | I0 | I1 | I0 | I1 | E1^**^ | I0 | I0 | I1 | I0 | I0 | I0 |  |  |
| 18 | I0 | I0 | I0 | I0 | I0 | I0 | S2 | I0 | I1 | E1^**^ | I0 | I0 | I1 | I0 | I0 | I0 |  |  |
| 19 | S1 | I0^*^ | I0 | I0 | I0 | I0 | E2 | I0 | I1 | E1^**^ | I0 | I0 | I1 | I0 | I0 | I0 |  |  |
| 20 | S1 | I0^*^ | S1^**^ | I0 | I0 | I0 | E2^*^ | I0 | I1 | E1^**^ | I0^*^ | S1^**^ | I1^*^ | I0 | S1^**^ | I0 |  |  |
| 21 | S1 | I0 | E1^**^ | I0 | I0 | I0 | E2 | I0 | I1 | E1^**^ | I0^*^ | E1^**^ | I1^*^ | S1^**^ | E1^**^ | I0 |  |  |
| 22 | S1 | I0 | E1^**^ | S1 | I0 | S1^**^ | E2 | I0 | S2 | E1^**^ | I0 | E1^**^ | I1 | E1^**^ | E1^**^ | I0 |  |  |
| 23 | S1 | I0 | E1^**^ | S1 | I0 | E1^**^ | I2 | S1 | S2 | E1^**^ | I0 | I1 | S2 | E1^**^ | E1^**^ | S1 |  |  |
| 24 | S1^**^ | S1^**^ | E1^**^ | S1^**^ | S1^**^ | E1^**^ | S3 | S1^**^ | S2^**^ | E1^**^ | I0^**^ | S2^**^ | S2^**^ | E1^**^ | I1^**^ | S1^**^ |  |  |
| 25 | S1^**^ | S1 | I1 | S1 | S1 | I1 | S3 | S1 | S2 | E1^**^ | I0 | S2 | S2 | I1 | I1 | S1 |  |  |
| 26 | S1^**^ | S1 | S2 | S1 | S1 | S2 | S3 | S1 | S2 | E1^**^ | S1 | S2 | S2 | S2 | S2 | S1 |  |  |
| 27 | S1^**^ | S1 | S2 | S1 | S1 | S2 | S3 | S1 | S2 | E1^**^ | S1 | S2 | S2 | S2 | S2 | S1 |  |  |
| 28 | S1^**^ | S1^**^ | S2^**^ | S1^**^ | S1^**^ | S2^**^ | S3 | S1^**^ | S2^**^ | E1^**^ | S1^*^ | S2^**^ | S2^**^ | S2^**^ | S2^**^ | S1^**^ |  |  |
| 29 | S1^**^ | S1 | S2 | S1 | S1 | S2 | S3 | S1 | S2 | E1^**^ | S1^*^ | S2 | S2 | S2 | S2 | S1 |  |  |
| 30 | S1^**^ | S1^**^ | S2^**^ | S1^**^ | S1^**^ | S2^**^ | S3 | S1^**^ | S2^**^ | E1^**^ | S1^**^ | S2^**^ | S2^**^ | S2^**^ | S2^**^ | S1^**^ |  |  |

See footnotes for Table 1.
