# Supplementary material for: Understanding the direct and indirect impacts of disease response phenotypes on chicken coccidiosis epidemiology: A modelling approach
Source: PLoS One. 2026 Mar 5;21(3):e0343712. doi: 10.1371/journal.pone.0343712 (PMC12962546; doi:10.1371/journal.pone.0343712)
Supplement: S3 Table — (DOCX) [file pone.0343712.s003.docx]

**Supplementary Table 3. States of Each Pair in Experiment III: 500-Oocyst Group (9 pairs).** Bird 525C had only 2 out of 30 records. Due to its lackness, the pair (of 546I and 525C) is excluded from the analysis).

|  | pair (with birds index) | | | | | | | | | | | | | | | | | |
| --- | --- | --- | --- | --- | --- | --- | --- | --- | --- | --- | --- | --- | --- | --- | --- | --- | --- | --- |
|  | 1 | | 2 | | 3 | | 4 | | 5 | | 6 | | 7 | | 8 | | 9 | |
| Day.start | 520I | 501C | 55I2 | 556C | 54I1 | 524C | 568I | 548C | 50I3 | 522C | 505I | 572C | 560I | 511C | 504I | 509C | 530I | 502C |
| 4 | E0 | S0^**^ | E0 | S0^**^ | E0 | S0^**^ | E0 | S0^**^ | E0 | S0^**^ | E0 | S0^**^ | I0 | S0^**^ | I0 | S0^**^ | I0 | S0^**^ |
| 5 | I0 | S0^**^ | I0 | S0^**^ | I0 | S0^**^ | I0 | S0^**^ | I0 | S0^**^ | I0 | S0^**^ | I0 | S0^**^ | I0 | E0^**^ | I0 | S0^**^ |
| 6 | I0 | E0^**^ | I0 | E0^**^ | I0 | E0^**^ | I0 | E0^**^ | I0 | E0^**^ | I0 | E0^**^ | I0 | E0^**^ | I0 | E0^**^ | I0 | E0^**^ |
| 7 | I0 | E0 | I0 | E0^**^ | I0 | E0 | I0 | E0^**^ | I0 | E0^**^ | I0 | E0^**^ | I0 | E0^**^ | I0 | E0^**^ | I0 | E0^**^ |
| 8 | I0 | E0 | I0 | E0 | I0 | E0 | I0 | E0 | I0 | E0 | I0 | E0 | I0 | E0 | I0 | E0 | I0 | E0 |
| 9 | I0^*^ | E0 | I0 | E0 | I0^*^ | E0 | I0 | E0 | I0 | E0 | I0 | E0 | I0 | E0 | I0 | I0 | I0^*^ | E0 |
| 10 | I0^*^ | I0 | I0 | I0 | I0 | I0 | I0 | I0 | I0 | I0 | I0 | I0 | I0 | I0 | I0 | I0 | I0 | I0 |
| 11 | I0 | I0 | I0 | I0 | I0 | I0 | I0 | I0 | I0 | I0 | I0 | I0 | I0 | I0 | I0 | I0 | I0 | I0 |
| 12 | I0 | I0 | I0 | I0 | I0 | I0 | I0 | I0 | I0 | I0 | I0 | I0 | I0 | I0 | I0 | I0 | I0 | I0 |
| 13 | I0 | I0 | I0 | I0 | I0 | I0 | I0 | I0 | I0 | I0 | I0 | I0 | I0 | I0 | I0 | I0 | I0 | I0 |
| 14 | I0 | I0 | I0 | I0 | I0 | I0 | I0 | I0 | I0 | I0 | I0 | I0 | I0 | I0 | I0 | I0 | I0 | I0 |
| 15 | I0 | I0 | I0 | I0^**^ | I0 | I0 | I0 | I0 | I0 | I0 | S1^**^ | I0 | I0 | I0 | I0 | I0 | I0 | I0 |
| 16 | I0 | I0 | I0 | I0 | I0 | I0 | I0^*^ | I0 | I0^*^ | I0 | E1^**^ | I0 | I0 | I0 | I0 | I0 | I0^*^ | I0 |
| 17 | I0 | I0 | I0 | I0 | I0 | I0 | I0^*^ | I0 | I0^*^ | I0 | E1^**^ | I0 | I0 | I0 | I0^*^ | I0 | I0^*^ | I0 |
| 18 | I0 | I0 | I0 | S1^**^ | I0^*^ | I0 | I0 | I0 | I0 | I0^*^ | I1 | I0 | I0 | I0 | I0 | S1^**^ | I0 | I0 |
| 19 | I0 | S1^**^ | I0^*^ | E1^**^ | I0 | I0 | I0 | I0 | I0 | I0 | S2 | I0 | I0 | I0 | I0 | E1^**^ | I0 | I0^*^ |
| 20 | I0 | E1^**^ | I0 | E1^**^ | I0^*^ | I0 | S1 | I0^*^ | I0 | I0 | S2 | I0 | I0 | I0 | S1 | E1^**^ | S1^**^ | I0 |
| 21 | I0^*^ | E1^**^ | I0 | I1 | I0 | I0^*^ | S1^**^ | I0^*^ | S1^**^ | S1^**^ | S2 | I0^*^ | S1^**^ | I0 | S1 | E1^**^ | E1^**^ | I0 |
| 22 | I0 | I1 | I0^*^ | S2 | S1^**^ | I0 | E1^**^ | I0 | E1^**^ | E1^**^ | S2 | I0^*^ | E1^**^ | I0^*^ | S1 | I1 | E1^**^ | S1 |
| 23 | I0 | I1^*^ | I0 | E2 | E1^**^ | I0 | E1^**^ | I0 | E1^**^ | E1^**^ | S2 | I0 | E1^**^ | I0 | S1 | S2 | I1 | S1 |
| 24 | S1^**^ | I1^**^ | I0^**^ | E2^**^ | E1^**^ | I0^**^ | E1^**^ | I0^**^ | E1^**^ | E1^**^ | S2^**^ | S1^**^ | E1^**^ | I0^**^ | S1^**^ | S2^**^ | I1^**^ | S1^**^ |
| 25 | S1 | I1 | I0 | I2 | I1 | I0 | E1^**^ | I0 | E1^**^ | I1 | S2 | S1 | I1 | I0 | S1 | S2 | I1 | S1 |
| 26 | S1 | I1 | I0 | I2 | I1 | I0 | I1 | I0 | I1 | I1 | S2 | S1 | I1 | S1 | S1 | S2 | I1 | S1 |
| 27 | S1 | I1 | S1 | I2 | S2 | I0 | S2 | I0 | I1 | S2 | S2 | S1 | I1 | S1 | S1 | S2 | I1 | S1 |
| 28 | S1^**^ | I1 | S1 | I2 | S2^**^ | I0 | S2^**^ | I0 | I1 | S2^**^ | S2^**^ | S1^**^ | I1 | S1^**^ | S1^**^ | S2^**^ | I1^**^ | S1^**^ |
| 29 | S1 | S2 | S1^**^ | S3 | S2 | S1 | S2 | S1 | S2 | S2 | S2 | S1 | S2 | S1 | S1 | S2 | I1 | S1 |
| 30 | S1^**^ | S2^**^ | S1 | S3^**^ | S2^**^ | S1^**^ | S2^**^ | S1^**^ | S2^**^ | S2^**^ | S2^**^ | S1^**^ | S2^**^ | S1^**^ | S1^**^ | S2^**^ | I1^**^ | S1^**^ |

See footnotes for Table 1.
