# Supplementary material for: Understanding the direct and indirect impacts of disease response phenotypes on chicken coccidiosis epidemiology: A modelling approach
Source: PLoS One. 2026 Mar 5;21(3):e0343712. doi: 10.1371/journal.pone.0343712 (PMC12962546; doi:10.1371/journal.pone.0343712)
Supplement: S4 Table — (DOCX) [file pone.0343712.s004.docx]

**Supplementary Table 4.** States of Each Pair in Experiment IV: 5000-Oocyst Group

|  | pair (with birds index) | | | | | | | | | | | |  |  |  |  |  |  |
| --- | --- | --- | --- | --- | --- | --- | --- | --- | --- | --- | --- | --- | --- | --- | --- | --- | --- | --- |
|  | 1 | | 2 | | 3 | | 4 | | 5 | | 6 | |  |  |  |  |  |  |
| Day.start | 827I | 803C | 764I | 756C | 31I1 | 823C | 74I1 | 847C | 310I | 839C | 795I | 857C |  |  |  |  |  |  |
| 4 | I0 | S0^**^ | E0 | S0^**^ | I0 | S0^**^ | I0 | S0^**^ | I0 | S0^**^ | I0 | S0^**^ |  |  |  |  |  |  |
| 5 | I0 | E0^**^ | I0 | S0^**^ | I0 | E0^**^ | I0 | S0^**^ | I0 | S0^**^ | I0 | S0^**^ |  |  |  |  |  |  |
| 6 | I0 | E0^**^ | I0 | E0^**^ | I0 | E0^**^ | I0 | E0^**^ | I0 | S0^**^ | I0 | E0^**^ |  |  |  |  |  |  |
| 7 | I0 | E0^**^ | I0 | E0^**^ | I0 | E0^**^ | I0 | E0^**^ | I0 | E0^**^ | I0 | E0^**^ |  |  |  |  |  |  |
| 8 | I0 | E0 | I0 | E0 | I0 | E0 | I0 | E0 | I0 | E0 | I0 | E0 |  |  |  |  |  |  |
| 9 | I0 | I0 | I0 | E0 | I0 | I0 | I0 | E0 | I0 | E0 | I0 | E0 |  |  |  |  |  |  |
| 10 | I0 | I0^*^ | I0 | I0 | I0 | I0 | I0 | I0 | S1^**^ | E0 | I0 | I0 |  |  |  |  |  |  |
| 11 | I0 | I0 | I0 | I0 | I0^**^ | I0 | I0 | I0 | E1^**^ | I0 | I0 | I0 |  |  |  |  |  |  |
| 12 | I0 | I0 | I0 | I0 | I0 | I0 | I0 | I0 | E1^**^ | I0 | I0 | I0 |  |  |  |  |  |  |
| 13 | I0 | I0 | I0 | I0 | I0^*^ | I0 | I0 | I0 | I1 | I0 | I0 | I0 |  |  |  |  |  |  |
| 14 | I0 | I0 | I0 | I0 | I0 | I0 | I0 | I0 | I1 | I0 | I0 | I0 |  |  |  |  |  |  |
| 15 | I0 | I0 | I0 | I0 | I0 | I0 | I0 | I0 | I1 | I0 | I0 | I0 |  |  |  |  |  |  |
| 16 | I0^*^ | I0 | S1^**^ | I0 | I0^*^ | I0 | I0^*^ | I0 | I1 | I0 | I0 | I0 |  |  |  |  |  |  |
| 17 | I0^*^ | I0 | E1^**^ | I0 | I0 | I0 | I0 | I0 | S2 | I0 | I0 | I0 |  |  |  |  |  |  |
| 18 | I0 | I0 | E1^**^ | I0 | I0 | I0 | I0 | I0 | S2 | S1^**^ | I0^**^ | I0 |  |  |  |  |  |  |
| 19 | S1^**^ | I0 | E1^**^ | I0 | S1 | I0 | I0^*^ | S1^**^ | S2 | E1^**^ | I0 | I0^*^ |  |  |  |  |  |  |
| 20 | E1^**^ | I0 | I1 | I0 | S1 | I0 | I0 | E1^**^ | S2 | E1^**^ | I0^*^ | I0 |  |  |  |  |  |  |
| 21 | E1^**^ | I0 | S2 | I0 | S1 | I0 | I0 | E1^**^ | S2 | E1^**^ | I0 | I0 |  |  |  |  |  |  |
| 22 | E1^**^ | I0 | S2^**^ | I0 | S1^**^ | I0^*^ | S1 | I1 | S2^**^ | I1 | I0^**^ | I0 |  |  |  |  |  |  |
| 23 | I1 | I0 | S2 | I0^*^ | S1 | I0 | S1 | S2 | S2^**^ | S2 | I0 | I0^*^ |  |  |  |  |  |  |
| 24 | S2 | S1 | S2^**^ | I0^*^ | S1^**^ | S1 | S1 | S2 | S2^**^ | S2 | I0 | I0 |  |  |  |  |  |  |
| 25 | S2 | S1 | S2^**^ | I0 | S1^**^ | S1 | S1^**^ | S2 | S2 | S2^**^ | I0 | I0 |  |  |  |  |  |  |
| 26 | S2^**^ | S1^**^ | S2^**^ | I0^**^ | S1^**^ | S1^**^ | S1^**^ | S2^**^ | S2^**^ | S2^**^ | I0^**^ | I0^*^ |  |  |  |  |  |  |

See footnotes for Table 1
