# Supplementary material for: Understanding the direct and indirect impacts of disease response phenotypes on chicken coccidiosis epidemiology: A modelling approach
Source: PLoS One. 2026 Mar 5;21(3):e0343712. doi: 10.1371/journal.pone.0343712 (PMC12962546; doi:10.1371/journal.pone.0343712)
Supplement: S5 Table — (DOCX) [file pone.0343712.s005.docx]

**Supplementary Table 5.** Comparison of flock performance between baseline scenario (all traits value = 0) and scenarios where the average individual trait value was set to 1 (or -1 for infectivity and susceptibility). Outcomes are presented as mean (standard deviation) with associated p-values from t-tests comparing each scenario to baseline. Simulations were performed with 500 replicates of 20 birds for each scenario.

|  | Baseline | Reduced infectivity  (Inf) | | Reduced susceptibility  (Sus) | | Increased recoverability  (Rec) | | Increased compensatory growth (CommpG) | | Increased tolerance  (Tol) | |
| --- | --- | --- | --- | --- | --- | --- | --- | --- | --- | --- | --- |
|  | Mean (sd) | Mean (sd) | p | Mean (sd) | p | Mean (sd) | p | Mean (sd) | p | Mean (sd) | p |
| Number of infections | 2.05 (0.32) | 1.68 (0.45) | <0.0001 | 1.68 (0.45) | <0.0001 | 1.86 (0.49) | <0.0001 | 2.04 (0.35) | 1 | 2.35 (0.41) | <0.0001 |
| Total duration of infection (day) | 14.17 (2.63) | 13.08 (3.95) | <0.0001 | 13.31 (4.09) | 0.00451 | 5.68 (1.80) | <0.0001 | 14.04 (2.89) | 1 | 17.45 (4.14) | <0.0001 |
| Mean prevalence | 0.25 (0.04) | 0.22 (0.06) | <0.0001 | 0.22 (0.07) | <0.0001 | 0.12 (0.04) | <0.0001 | 0.25 (0.05) | 1 | 0.27 (0.06) | <0.0001 |
| Death rate (%) | 0.21 (0.11) | 0.15 (0.10) | <0.0001 | 0.15 (0.10) | <0.0001 | 0.01 (0.03) | <0.0001 | 0.20 (0.11) | 1 | 0.00 (0.00) | <0.0001 |
| Mean final weight (g) | 1931.3 (22.3) | 1937.5 (31.4) | 0.0185 | 1935.9 (32.5) | 0.5079 | 1990.6 (14.7) | <0.0001 | 1944.0 (23.2) | <0.0001 | 1980.1 (14.2) | <0.0001 |
| Sandard deviation of final weight (g) | 54.20 (13.68) | 57.57 (20.31) | 0.1175 | 58.20 (19.96) | 0.0128 | 31.40 (11.35) | <0.0001 | 50.64 (15.80) | 0.0079 | 36.82 (11.15) | <0.0001 |
| Maximal infectious load in environment | 33.97 (5.98) | 10.68 (3.01) | <0.0001 | 28.85 (8.50) | <0.0001 | 20.39 (6.27) | <0.0001 | 33.45 (6.30) | 1 | 34.11 (6.80) | 1 |
| Mean infectious load in environment | 11.47 (1.89) | 3.70 (1.07) | <0.0001 | 10.13 (3.00) | <0.0001 | 5.73 (1.74) | <0.0001 | 11.40 (2.06) | 1 | 13.24 (2.74) | <0.0001 |
| Proportion of final meet loss | 0.25 (0.11) | 0.19 (0.10) | <0.0001 | 0.20 (0.10) | <0.0001 | 0.03 (0.03) | <0.0001 | 0.24 (0.11) | 0.2538 | 0.03 (0.01) | <0.0001 |
| Sum of weight loss (g) | 103110 (20746) | 95564 (28640) | 0.0001 | 95899 (29159) | 0.0004 | 56989 (17918) | <0.0001 | 97751 (21608) | 0.0037 | 59900 (14136) | <0.0001 |
| Area of resilience pathway | 256.28 (73.49) | 66.57 (26.55) | <0.0001 | 182.27 (75.38) | <0.0001 | 68.53 (31.96) | <0.0001 | 246.15 (72.65) | 1 | 105.07 (32.33) | <0.0001 |
