# Supplementary material for: Understanding the direct and indirect impacts of disease response phenotypes on chicken coccidiosis epidemiology: A modelling approach
Source: PLoS One. 2026 Mar 5;21(3):e0343712. doi: 10.1371/journal.pone.0343712 (PMC12962546; doi:10.1371/journal.pone.0343712)
Supplement: S6 Table — The direct effect of the trait is estimated by considering an individual with a favorable trait among individuals with baseline traits. The indirect effect is estimated by considering an individual with baseline trait among individual with favorable traits. Values are presented as mean (standard deviation), with p-values comparing each scenario with baseline. Simulations were performed with 500 replicates of 20 birds for each scenario. (DOCX) [file pone.0343712.s006.docx]

**Supplementary Table 6.** Comparison of one animal performance between baseline scenario and alternative scenarios (Inf: decreased infectivity, Sus: decreased susceptibility, Rec: increased recoverability, CompG: increased compensatory growth, Tol: increased tolerance). The direct effect of the trait is estimated by considering an individual with a favorable trait among individuals with neutral traits. The indirect effect is estimated by considering an individual with neutral trait among individual with favorable traits. Values are presented as mean (standard deviation), with p-values comparing each scenario with baseline. Simulations were performed with 500 replicates of 20 birds for each scenario.

|  | Baseline | Susceptibility (Sus) | | | | Infectivity (Inf) | | | |
| --- | --- | --- | --- | --- | --- | --- | --- | --- | --- |
|  |  | Direct | | Indirect | | Direct | | Indirect | |
|  | mean(sd) | mean(sd) | p_value | mean(sd) | p_value | mean(sd) | p_value | mean(sd) | p_value |
| Number of infection (n) | 2.04 (0.74) | 1.72 (0.66) | <0.0001 | 2.06 (0.83) | 1 | 2.02 (0.78) | 1 | 1.66 (0.76) | <0.0001 |
| Total duration of infection (days) | 14.41 (7.16) | 13.01 (7.44) | 0.1662 | 14.41 (7.73) | 1 | 14.25 (7.41) | 1 | 13.21 (8.56) | 1 |
| Time before first infection (days) | 7.47 (4.59) | 8.91 (4.6) | 0.0001 | 7.53 (4.41) | 1 | 6.98 (4.32) | 1 | 10.77 (6.24) | <0.0001 |
| Final weight (g) | 1930.44 (59.48) | 1940.97 (58.3) | 0.7968 | 1928.75 (63.27) | 1 | 1929.55 (61.04) | 1 | 1937.41 (70.28) | 1 |
| Maximal weight deviation (%) | 13.26 (5.63) | 11.76 (5.94) | 0.0032 | 12.64 (5.96) | 1 | 13.06 (5.6) | 1 | 11.1 (6.25) | <0.0001 |
| Sum of weight loss (g) | 464.84 (240.42) | 416.74 (237.6) | 0.1055 | 461.66 (262.24) | 1 | 474.76 (251.21) | 1 | 395.5 (244.82) | 0.0005 |
| Death (yes/no) | 0.22 (0.41) | 0.16 (0.37) | 1 | 0.19 (0.4) | 1 | 0.19 (0.39) | 1 | 0.15 (0.36) | 0.2294 |

|  | Compensatory growth (CompG) | | | | Recoverability (Rec) | | | | Tolerance (Tol) | | | |
| --- | --- | --- | --- | --- | --- | --- | --- | --- | --- | --- | --- | --- |
|  | Direct | | Indirect | | Direct | | Indirect | | Direct | | Indirect | |
|  | mean(sd) | p_value | mean(sd) | p_value | mean(sd) | p_value | mean(sd) | p_value | mean(sd) | p_value | mean(sd) | p_value |
| Number of infection (n) | 2.03 (0.77) | 1 | 2.08 (0.73) | 1 | 2.44 (0.67) | <0.0001 | 1.57 (0.71) | <0.0001 | 2.08 (0.7) | 1 | 2.21 (0.84) | 0.0326 |
| Total duration of infection (days) | 14.08 (7.42) | 1 | 14.28 (7.21) | 1 | 6.4 (4.5) | <0.0001 | 12.89 (7.88) | 0.0986 | 15.85 (11.48) | 1 | 14.29 (7.62) | 1 |
| Time before first infection (days) | 7.45 (4.59) | 1 | 7.31 (4.57) | 1 | 7.31 (4.67) | 1 | 7.88 (4.88) | 1 | 7.33 (4.67) | 1 | 7.41 (4.51) | 1 |
| Final weight (g) | 1942 (58) | 0.2179 | 1925 (62) | 1 | 1984 (35) | <0.0001 | 1942 (61) | 0.2866 | 1986 (37) | <0.0001 | 1929 (63) | 1 |
| Maximal weight deviation (%) | 12.88 (5.7) | 1 | 13.19 (5.47) | 1 | 7.01 (4.63) | <0.0001 | 12.24 (6.23) | 0.4592 | 4.82 (2.59) | <0.0001 | 12.69 (5.71) | 1 |
| Sum of weight loss (g) | 435 (236) | 1 | 491 (256) | 1 | 292 (202) | <0.0001 | 432 (255) | 1 | 228 (140) | <0.0001 | 465 (251) | 1 |
| Death (yes/no) | 0.21 (0.41) | 1 | 0.17 (0.38) | 1 | 0.01 (0.12) | <0.0001 | 0.17 (0.37) | 1 | 0 (0) | <0.0001 | 0.19 (0.39) | 1 |
