# Supplementary figures and images for: Understanding the direct and indirect impacts of disease response phenotypes on chicken coccidiosis epidemiology: A modelling approach
Source: PLoS One. 2026 Mar 5;21(3):e0343712. doi: 10.1371/journal.pone.0343712 (PMC12962546; doi:10.1371/journal.pone.0343712)

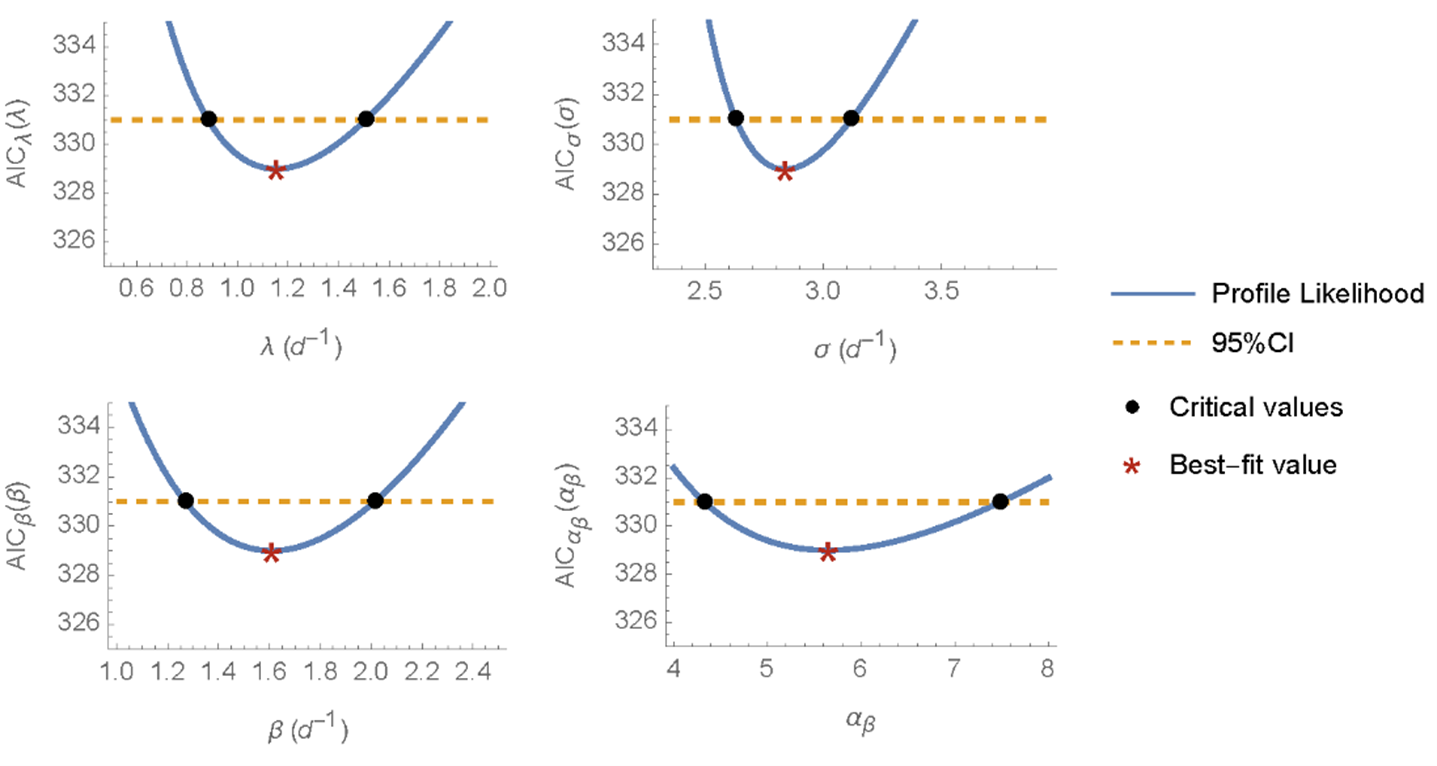

Supplement: S1 Fig — The x-axis shows parameter values, and the y-axis shows AIC values. The solid line represents profile likelihood AIC values, with the lowest point (red star) indicating the best estimate. Black dots mark critical values for the 95% confidence interval, defined as the minimum AIC plus 2 (dashed line). (PNG) [file pone.0343712.s007.png]

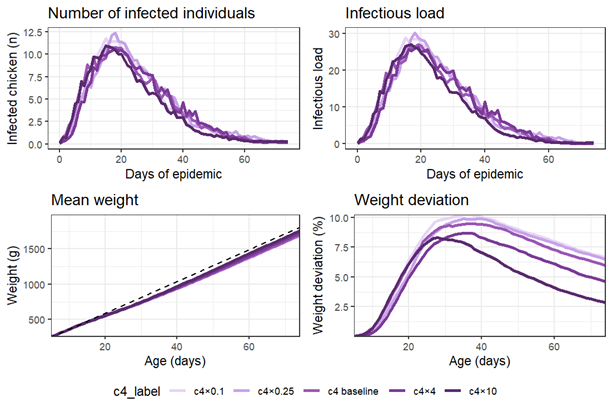

Supplement: S2 Fig — For each c4 value, we simulated all six trait scenarios (baseline, lower susceptibility, lower infectivity, higher recoverability, improved compensatory growth, and improved tolerance) with 20 replications each, yielding 600 simulations total. The figure shows that epidemic dynamics (number of infected individuals, infectious load, mean weight trajectories) remain nearly identical across all c4 values. Weight deviation patterns scale predictably with c4: higher values produce faster post-recovery weight gain (steeper decline in weight deviation after peak infection), but maximum weight deviation during acute infection remains similar across scenarios. Importantly, trait rankings remain stable across the entire parameter space for epidemic-related outcomes. (PNG) [file pone.0343712.s008.png]

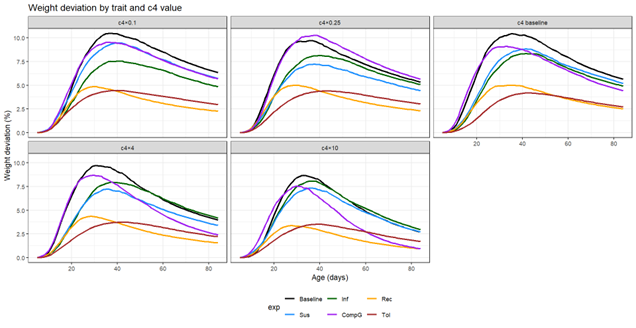

Supplement: S3 Fig — The relative performance of different trait improvement strategies shows consistent patterns across most of the c4 range. However, at the highest compensatory growth value tested (c4 × 10), improvements in compensatory growth show enhanced benefits on final weight and cumulative weight loss relative to susceptibility and infectivity improvements, indicating that the relative value of trait improvement strategies may shift under conditions of exceptionally rapid compensatory growth capacity. (PNG) [file pone.0343712.s009.png]
