# Supplementary material for: Understanding the direct and indirect impacts of disease response phenotypes on chicken coccidiosis epidemiology: A modelling approach
Source: PLoS One. 2026 Mar 5;21(3):e0343712. doi: 10.1371/journal.pone.0343712 (PMC12962546; doi:10.1371/journal.pone.0343712)
Supplement: Attachment — Submitted filename: Suggestions.pdf [file pone.0343712.s011.pdf]

Suggestions

The manuscript is technically sophisticated and addresses a critical gap in infectious disease modeling for livestock. However, the conclusions regarding compensatory growth are weakened by the use of arbitrary parameter values and a lack of sensitivity analysis for these specific variables.

Major Weaknesses and Critical Issues

1. Parameters for compensatory growth (p) and mortality rates (μ) were set arbitrarily due to a lack of specific literature data. Because these traits are core to the paper's conclusions regarding resilience, the results for scenario "CompG" and "Tol" may be artifacts of these specific values rather than generalizable biological truths.
2. The model is calibrated exclusively on *E. acervulina* data. Other species like *E. tenella* or *E. maxima* cause significantly higher mortality and different growth depression patterns. The conclusions may not hold for the most economically damaging forms of coccidiosis. Fix by adding a discussion/simulation of "high-virulence" parameters.
3. The model assumes a constant decay rate (λ) for oocysts, whereas oocyst survival is highly dependent on environmental humidity and temperature. In real-world poultry houses, pathogen accumulation is non-linear and affected by litter quality, which could drastically change the indirect benefits of lower infectivity.
4. In Table 1 and Equation [iv], the authors define the infection probability. Please clarify if N is the total population or the number of susceptible individuals, as this affects the density-dependence vs. frequency-dependence assumptions of the model.
5. The mortality trigger G* is set at 0.8 (20% weight loss). While Table 4 shows deviations up to 28.9%, mortality in coccidiosis is often due to acute intestinal hemorrhage (for *E. tenella*) rather than just weight loss. The authors should justify why weight loss is a sufficient proxy for mortality across different infection intensities.
6. On lines 164-168, growth is modeled proportional to the difference between current and theoretical weight. I suggest the authors test if a leaky recovery model, where some growth potential is permanently lost would change the resilience loop area significantly.
7. In Fig 5, the units of L(t) are scaled and difficult to compare directly with oocyst counts. A dual-axis or normalized plot would be more intuitive.
8. Coccidiosis is a parasitic disease... caused by... Eimeria. Since this is mentioned in the abstract, the opening of the introduction is slightly repetitive.

9. Assumed exposed period of 4 days. Provide a brief range if available for other species.
10. Parameter sigma (Shedding rate) is listed as 2.84 d^{-1}. Please ensure the units are consistent with oocysts per unit time or infectious load units.

Reviewer Suggestion on Requested References:

As a reviewer, I have examined additional references suggested for inclusion.

In the Discussion (Lines 1053–1056), where you discuss how vaccines enhance recoverability and reduce susceptibility, the following works provide excellent comparative context for poultry immunoprophylaxis:

Sajid, S., ur Rahman, S., & Mohin, M. (2024). Development of egg yolk-based polyclonal antibodies and immunoprophylactic potential of antigen-antibody complex against infectious bursal disease. *Veterinary and Animal Science*, *23*, 100326.

Sajid, S., ur Rahman, S., & Mohsin, M. (2022). IgY: a key isotype and promising antibody for the immunoprophylaxis therapy of infectious bursal disease virus infections. *Microbiology and Biotechnology Letters*, *50*(3), 430-435.

Sajid, S., & Mohsin, M. (2022). Development of an Immune Complex Vaccine against Infectious Bursal Disease Virus and its Potential Response in Poultry Birds. *Iranian Journal of Medical Microbiology*, *16*(6), 528-536.

Introduction (Line 249), you mention nutrition as a factor influencing host traits. This study on turnip powder in cereal blends could support the nutritional intervention aspect:

Toor, I. F., Sajid, S., Akmal, A., Abidin, Z. U., Fatima, Z., Althawab, S. A., ... & Alsulami, T. (2025). Nutritional Evaluation of Turnip Powder in Cereal Blends: A Study on Wheat, Oats, and Turnips. *Food Science & Nutrition*, *13*(5), e70157.

In the Discussion (Lines 1060–1063), you mention gut microbiota as a target for modifying host responses. This work on *Lactobacillus* could support that specific point:

Farzand, I., ur Rahman, S., Sajid, S., & Nayab, S. (2020). *Evaluation of modified MRS media for the selective enumeration of Lactobacillus casei. Pure and Applied Biology. Vol. 10, Issue 1, pp194-198*.

The works on *Infectious Bursal Disease Virus (IBDV)* and *Mycoplasma* demonstrate the variability of host responses in Pakistani broiler populations. These could be used in the Discussion when you talk about the need for local epidemiological data.

Sajid, S., Rahman, S. U., Mohsin Gilani, M., Sindhu, Z. U. D., Ali, M. B., Hedfi, A., ... & Mahmood, S. (2021). Molecular characterization and demographic study on infectious bursal disease virus in faisalabad district. *Plos one*, *16*(8), e0254605.

The following papers could strengthen your Discussion regarding alternative intervention strategies:

Chen, J., Chen, F., Peng, S., Ou, Y., He, B., Li, Y., & Lin, Q. (2022). Effects of Artemisia argyi Powder on Egg Quality, Antioxidant Capacity, and Intestinal Development of Roman Laying Hens. Frontiers in physiology, 13, 902568. https://doi.org/10.3389/fphys.2022.902568

Hassan, F. U., Liu, C., Mehboob, M., Bilal, R. M., Arain, M. A., Siddique, F., Chen, F., Li, Y., Zhang, J., Shi, P., Lv, B., & Lin, Q. (2023). Potential of dietary hemp and cannabinoids to modulate immune response to enhance health and performance in animals: opportunities and challenges. Frontiers in immunology, 14, 1285052. https://doi.org/10.3389/fimmu.2023.1285052

Liu, H., Bing, P., Zhang, M., Tian, G., Ma, J., Li, H., Bao, M., He, K., He, J., He, B., & Yang, J. (2023). MNNMDA: Predicting human microbe-disease association via a method to minimize matrix nuclear norm. Computational and structural biotechnology journal, 21, 1414–1423. https://doi.org/10.1016/j.csbj.2022.12.053
