# Supplementary material for: Understanding the direct and indirect impacts of disease response phenotypes on chicken coccidiosis epidemiology: A modelling approach
Source: PLoS One. 2026 Mar 5;21(3):e0343712. doi: 10.1371/journal.pone.0343712 (PMC12962546; doi:10.1371/journal.pone.0343712)
Supplement: Attachment — Submitted filename: Response to reviewers.docx [file pone.0343712.s010.docx]

We thank the editor and reviewer for their thorough review and constructive comments, which have significantly improved the quality and clarity of our manuscript. We have carefully addressed all comments point-by-point below and made the corresponding changes throughout the revised manuscript. We believe these revisions have strengthened our work and we are grateful for the valuable feedback.

Journal Requirements:

When submitting your revision, we need you to address these additional requirements.

1. Please ensure that your manuscript meets PLOS ONE's style requirements, including those for file naming. The PLOS ONE style templates can be found at

<https://journals.plos.org/plosone/s/file?id=wjVg/PLOSOne_formatting_sample_main_body.pdf> and

[https://journals.plos.org/plosone/s/file?id=ba62/PLOSOne_formatting_sample_title_authors_affiliations.pdf](https://track.editorialmanager.com/CL0/https:%2F%2Fjournals.plos.org%2Fplosone%2Fs%2Ffile%3Fid=ba62%2FPLOSOne_formatting_sample_title_authors_affiliations.pdf/1/010f019a7c186fd3-4ff23776-e51d-4fd7-964d-519a7d58570d-000000/yA0zDTeMJ3QPM3BMV6MyD_6AfSjJt76VCyL3DOi1sHU=237)

**Response**: We have updated the entire manuscript to meet PLOS ONE style requirements. Specifically:

•	All figure files follow the naming convention (Fig1.tif, Fig2.tif, etc.)

•	Title and authors are formatted according to the PLOS ONE template

•	All heading levels are correct (Level 1, 2, 3)

•	All references follow PLOS ONE citation style

2. Please note that PLOS One has specific guidelines on code sharing for submissions in which author-generated code underpins the findings in the manuscript. In these cases, we expect all author-generated code to be made available without restrictions upon publication of the work. Please review our guidelines at <https://journals.plos.org/plosone/s/materials-and-software-sharing#loc-sharing-code> and ensure that your code is shared in a way that follows best practice and facilitates reproducibility and reuse.

**Response**: We have deposited all author-generated code to GitHub, a publicly accessible repository that issues DOIs. The code is available at:

<https://github.com/MarieIthurbi/coccidiosis_simulation>

3. Thank you for stating the following financial disclosure: ”European Union's Horizon Europe Project 10113646 EUPAHW.” Please state what role the funders took in the study.  If the funders had no role, please state: "The funders had no role in study design, data collection and analysis, decision to publish, or preparation of the manuscript." If this statement is not correct you must amend it as needed. Please include this amended Role of Funder statement in your cover letter; we will change the online submission form on your behalf.

**Response**: We have amended our funding statement. The complete statement now reads: "This work was co-funded by the European Union's Horizon Europe Project 10113646 EUPAHW. The funders had no role in study design, data collection and analysis, decision to publish, or preparation of the manuscript." This statement has been included in our cover letter and should replace the current funding statement in the submission system.

4. Thank you for stating the following in the Acknowledgments Section of your manuscript: “This work was co-funded by the European Union's Horizon Europe Project 10113646 745 EUPAHW.” We note that you have provided funding information that is not currently declared in your Funding Statement. However, funding information should not appear in the Acknowledgments section or other areas of your manuscript. We will only publish funding information present in the Funding Statement section of the online submission form. Please remove any funding-related text from the manuscript and let us know how you would like to update your Funding Statement. Currently, your Funding Statement reads as follows: ”European Union's Horizon Europe Project 10113646 EUPAHW.” Please include your amended statements within your cover letter; we will change the online submission form on your behalf.

**Response**: We understand that funding information should appear only in the Funding Statement section. We have removed all funding-related text from the Acknowledgments section of the manuscript.

5. We note you have included a table to which you do not refer in the text of your manuscript. Please ensure that you refer to Table 2 in your text; if accepted, production will need this reference to link the reader to the Table.

**Response**: We have added a reference to Table 2 on line 144 of the revised manuscript, ensuring all tables are properly cited in the text.

Additional Editor Comments: The authors needs to revise the manuscript in accordance to the comments of the reviewers, with special attention to add more details in the section on materials and methods
**Response: We thank the editor for these valuable recommendations. We have carefully revised the manuscript in accordance with all reviewer comments.**

Reviewers' comments

1. Is the manuscript technically sound, and do the data support the conclusions?

The manuscript must describe a technically sound piece of scientific research with data that supports the conclusions. Experiments must have been conducted rigorously, with appropriate controls, replication, and sample sizes. The conclusions must be drawn appropriately based on the data presented.

Reviewer #1: Yes

2. Has the statistical analysis been performed appropriately and rigorously?

Reviewer #1: Yes

3. Have the authors made all data underlying the findings in their manuscript fully available?

The [PLOS Data policy](http://track.editorialmanager.com/CL0/http:%2F%2Fwww.plosone.org%2Fstatic%2Fpolicies.action%23sharing/1/010f019a7c186fd3-4ff23776-e51d-4fd7-964d-519a7d58570d-000000/1Ad-r2BPnG2cteqxwoAM8jmE5dqbZ1rcFfNstS16NpM=237) requires authors to make all data underlying the findings described in their manuscript fully available without restriction, with rare exception (please refer to the Data Availability Statement in the manuscript PDF file). The data should be provided as part of the manuscript or its supporting information, or deposited to a public repository. For example, in addition to summary statistics, the data points behind means, medians and variance measures should be available. If there are restrictions on publicly sharing data—e.g. participant privacy or use of data from a third party—those must be specified.

Reviewer #1: Yes

4. Is the manuscript presented in an intelligible fashion and written in standard English?

PLOS ONE does not copyedit accepted manuscripts, so the language in submitted articles must be clear, correct, and unambiguous. Any typographical or grammatical errors should be corrected at revision, so please note any specific errors here.

Reviewer #1: Yes

5. Review Comments to the Author

Reviewer #1: The model proposed by the authors appears intriguing and would contribute significantly to the treatment of avian coccidiosis. However, the work contains some errors that would need to be corrected to improve the manuscript. It is important for the authors to remember that every time the scientific name of a species (genus or species) is mentioned, it must be written in italics, in accordance with international standards for taxonomic nomenclature.

**Response**: Corrected. All scientific names (genus and species) are now properly italicized in accordance with international standards for taxonomic nomenclature. We reviewed every instance of species names including Eimeria spp. and other organisms mentioned in the text.

The manuscript often mixes the figure legends with the informative text. For example, in the introduction section, the paragraph between lines 53 and 67 should not be there; the figure legends should be at the end of the manuscript, after the reference list.

**Response**: Corrected. All figure legends have been moved to the end of the manuscript, after the References section, as per PLOS ONE requirements.

Some information in the introduction lacks bibliographic support (e.g., line 73: ...and 5- compensatory growth occurring after the infection ends.)

**Response**: We thank the reviewer for this helpful observation. We agree that several statements in the introduction required additional bibliographic support. We have now added several references throughout the introduction to address this issue. Specifically, we have incorporated references 3 and 4 (Doeschl-Wilson et al., 2021; Pooley et al., 2020) to support our statements on disease resilience and individual variation in infection dynamics. We have also added references 7, 8, 11, 12, and 13 (Henken et al., 1994; Martin and Sauvant, 2010; Yun et al., 2000; Voeten et al., 1988) to better document the mechanisms of coccidiosis infection, immune responses, and compensatory growth effects on broiler performance. These additions provide a more comprehensive and well-supported framework for our study.

Some paragraphs are excessively long, which makes it difficult to understand the message.

**Response**: Corrected. We revised the manuscript to improve readability by breaking up long paragraphs.

Some citations do not conform to the journal's style (e.g., line 180: Voeten, 1989; line 197: Velkers (2010), et al.). Please review and correct this.

**Response**: Corrected. We have reviewed and corrected all citations to conform to PLOS ONE style.
